# Professor Adib Jatene, a youth of 80 years^*^

**DOI:** 10.1590/S1516-31802010000200001

**Published:** 2010-03-04

**Affiliations:** I Attending surgeon in the Division of Thoracic Surgery, Instituto do Coração (Incor), Hospital das Clínicas (HC), Faculdade de Medicina da Universidade de São Paulo (FMUSP), and associate professor, Department of Cardiopneumonology, Faculdade de Medicina da Universidade de São Paulo (FMUSP), São Paulo, Brazil.; II Chairman of the Board of Directors, Instituto do Coração (Incor), Hospital das Clínicas (HC), Faculdade de Medicina da Universidade de São Paulo (FMUSP), and titular professor of the Discipline of Cardiovascular Surgery, Faculdade de Medicina da Universidade de São Paulo (FMUSP), São Paulo, Brazil.

On reaching 80 years of age, Professor Adib Jatene has received deserved homage from the people of the state of São Paulo through their legitimate representatives in the State Legislature. Although few people achieve this public renown, there is another sphere of recognition of great importance: that of his professional peers. Even though professor Jatene's importance within the field of medicine has long been a matter of consensus, it is never too much to repeat what everyone within more restricted settings already knows.

Writing about Professor Jatene is to propose the challenge of looking into a human being of uncommon qualities: the personality traits of a brilliant thinker and tireless worker and innovator in the search for answers to crucial questions for the society of his time. In fact, for this octogenarian youth — recognized as an admirable scientist, surgeon and teacher, an audacious public administrator and the head of an exemplary family of four children (three of them also physicians) and ten grandchildren — the time is always now, because it is in the present that human beings become complete in all their facets. And it is precisely the human being that has been the central focus of Professor Jatene's existence. Although we do not intend in this article to cover the entirety of the unparalleled figure that Professor Jatene is, we will take on this challenge as a tribute to the gratifying opportunity that we have had of being at his side in the Heart Institute (InCor) of Hospital das Clínicas (HC) and in the School of Medicine of the University of São Paulo (FMUSP), for more than four decades.

The list of activities in the professor's extensive curriculum vitae includes technical innovations within bioengineering; surgical techniques that bear his own name and which are recognized internationally; setting up research and teaching laboratories in important medical centers; and activities such as administration of university schools, hospitals and foundations, and holding public office of the highest importance, such as Health Secretary of São Paulo and national Minister of Health. Even after the length of time that Professor Jatene has been frequenting classrooms, wards and surgical theaters, it is still wonderful to watch him, in the fullness of his 80 years, debating in the battlefields of public health, with the jovial spirit characteristic of those who are not content with the commonplace. The banner that he has always held advocates a comprehensive and equitable healthcare system that ensures wellbeing for all. The flame of his ideas always burns brightly when the topic is proposals for bold healthcare projects that bring quality multiprofessional medical care to all citizens, independent of their economic and social situation. And also for development of tools that help physicians to restore their patients’ health and wellbeing. Professor Jatene has dedicated himself, body and soul, to this mission not for party-political or philanthropic reasons or with a view to public office: he has done this because of the philosophical and humanistic view that has sustained his life, in which the essence of human beings is their wholeness, without differentiation through issues as random as social and economic condition.

It is precisely this compassionate view of his fellow human beings that has made Professor Adib Jatene an example among an intellectual elite that has a true commitment to the country and its citizens. His incessant efforts and search for the perfection that is possible in all acts is no more than his ever-present aspiration to help people is distress to feel better. On many occasions, in reflective conversations, Professor Jatene has cited Mother Teresa of Calcutta to explain the light that should guide intelligence in seeking solutions for the widest diversity of issues within human life: “Without faith, there is no love; Without love, there is no gift of self; Without the gift of self, there is no help for people in distress”.

Only full gifting to the common good can explain the Professor's unusual path, starting from his childhood in Xapuri, in the state of Acre, where he was born on June 4, 1929, and also where he lost his father when he was still only two years old, a victim of fulminating disease acquired in the forest. It would not be unusual for a young man from the backwoods of the country, without a father, to follow the path of so many other millions of Brazilians who, despite their valor in the daily struggle for survival, remain anonymous. But the history of this man, who was guided by a passion for knowledge and for the human being, followed another path.

After journeying around Brazil, with his family, until reaching Uberlândia, in the state of Gerais, the young Adib Jatene came to São Paulo, where he studied sciences at the renowned Colégio Bandeirantes. At that time, his aspiration to be an engineer underwent a surprising shift to medicine.

After graduating in medicine in 1953, from FMUSP, Professor Jatene did all of his specialized training in Brazil, at HC-FMUSP, under the guidance of Professor Euryclides de Jesus Zerbini, with whom he had started to work in 1951, while still an undergraduate student. He remained with Professor Zerbini until 1955 and subsequently from 1958 to 1962, when he then began his path towards leadership.

Between 1955 and 1957, he worked in Uberaba, where he introduced thoracic surgery into the region. There, he constructed his first model for an artificial heart and lung, thus retrieving his engineering spirit. Although still a young physician, he was a professor of topographical anatomy at the School of Medicine of the Triângulo Mineiro.

From 1958 to 1961, he was a surgeon at HC-FMUSP and at the Dante Pazzanese Institute of Cardiology, of the Health Department of the State of São Paulo. During this period, he organized an experimental and research laboratory at HC, in which he developed and constructed the first artificial heart-lung apparatus. This laboratory evolved into the prestigious Bioengineering Division of InCor.

In 1961, he left HC, to take up a permanent position in the Dante Pazzanese Institute of Cardiology. There, he was successively head of the Experimental and Research Laboratory and head of the Surgical Section, and subsequently medical director and general director. Simultaneously, he organized the Bioengineering Workshop, where a variety of apparatus and devices were studied, planned and developed, some of them original inventions. The workshop became the Technical Center for Research and Experiments in 1982.

Since 1977, he has been the general director of the Heart Hospital of the Syrian Sanitarium Association. He was a founder-member and the first president of the Brazilian Cardiology Society of the State of São Paulo, from 1977 to 1979, and he was Health Secretary of São Paulo from 1979 to 1982. Until March 1983, he was president of the Special Commission for implementing the primary healthcare system in the metropolitan region of São Paulo, for which the plan was drawn up and implementation began during his administration at the Health Department of São Paulo. He negotiated internal and external resources in order to ensure continuity for the project. Throughout this period, his medical activities continued uninterrupted.

In 1980, he founded the National Council of Health Secretaries (Conass), for which he was the first president. In 1983, with the retirement of Professor Zerbini, he went through a competitive selection process for the position of titular professor of Thoracic Surgery at FMUSP. He achieved the position and held it, together with the position of director of InCor, until 1999, when he had to retire compulsorily on completing 70 years of age.

In all, Professor Jatene has received many titles and representative positions within society: president of the Department of Cardiovascular Surgery of the Brazilian Cardiology Society (1985), honorary member of the American Association for Thoracic Surgery (1984), founder-member and president of the Brazilian Society of Cardiovascular Surgery (1984/1985), president of the Brazilian Cardiology Society (1985/1987) and president of the International Society for Cardiovascular Surgery (1985/1987). He was also a member of the Commission of Medical Education Specialists of the Ministry of Education (1986/1990), the National Health Board (1986/1992) and the Regional Medical Council of the State of São Paulo (1988/1992). In 1989, he was elected a titular member of the National Academy of Medicine and, in October 1990, director of FMUSP, for a four-year period.

He was a member of the National Social Security Board and of the Federal Education Board and Minister of Health, for eight months during the Collor presidency, and for 22 months during the Fernando Henrique Cardoso presidency. Within public administration, he introduced criticisms into the accounts processing system; he implemented integrated scheduling; he created the minimum standard for primary care; he presided over the ninth and tenth National Health Conferences; he drew up the basic operating standard 1/96, which consolidated the Brazilian National Health System (*Sistema Único de Saúde*, SUS); and gave great emphasis to the Family Health and Community Agent programs. He fought for linkage of resources and, in a record time, negotiated the Program for Reinforcement of SUS Reorganization (REFORSUS) with the World Bank and the Inter-American Development Bank. The resources from this were distributed to several states according to their populations.

He has authored and co-authored around 700 scientific studies published in Brazilian and foreign journals and is a member of 32 scientific societies in various regions of the world. He has received 178 titles and honors from more than 10 countries.

Among his many original contributions within the field of bioengineering are bubble and membrane oxygenators and tilting-disc valves, for which he holds patents. These pieces of equipment are being produced industrially under license and are used in Brazil and abroad.

Professor Jatene has also made important contributions within the fields of myocardial revascularization surgery and congenital heart disease surgery. He described the technique to correct transposition of the fundamental great vessels that today is known as the Jatene Procedure, which has been successfully used in many heart surgery services around the world. The surgical teams that he has led since 1962 have now carried out more than 80,000 operations. Many services in Brazil and through South America are led by surgeons who were trained under his guidance.

One of the campaigns taken up by Professor Jatene within his professional career has been defense of the quality of medical education. He was one of the people responsible for attempting to slow down the disorderly expansion of medical schools, since this would put at risk the performance of future physicians. During the 1980s, he participated actively in the discussions of the Medical Education Commission of the Ministry of Education. He proposed that only schools that had sufficient structure for student training should be able to offer the course, i.e. that these educational institutions would need to have had a hospital and outpatient medical complex in operation for at least two years, functioning as a regional referral center.

And because the professor's time is always the present, he continues to be fully active as a physician, an administrator (as the director of the Heart Hospital) and an advisor to the Ministry of Health regarding regulatory matters for medical schools.

His heart, firm and strong at 80 years of age, continues to have the same passion that the young physician had for knowledge and for what he has in his power to transform people's lives into a better world.

The good examples are eternal.

Professor Adib Jatene continues to be a great inspiration for all of us.

